# Identification of a miRNA Panel with a Potential Determinant Role in Patients Suffering from Periodontitis

**DOI:** 10.3390/cimb45030145

**Published:** 2023-03-08

**Authors:** Oana Baru, Lajos Raduly, Cecilia Bica, Paul Chiroi, Liviuta Budisan, Nikolay Mehterov, Cristina Ciocan, Laura Ancuta Pop, Smaranda Buduru, Cornelia Braicu, Mandra Badea, Ioana Berindan-Neagoe

**Affiliations:** 1 Department of Preventive Dentistry, Faculty of Dental Medicine, Iuliu Hatieganu University of Medicine and Pharmacy, 400083 Cluj-Napoca, Romania; 2 Research Center for Functional Genomics, Biomedicine and Translational Medicine, Iuliu Hatieganu University of Medicine and Pharmacy, 400015 Cluj-Napoca, Romania; 3 Stomestet Stomatology Clinic, Calea Manastur 68A Street, 400658 Cluj-Napoca, Romania; 4 Department of Medical Biology, Medical University of Plovdiv, 4002 Plovdiv, Bulgaria; 5 Research Institute, Medical University of Plovdiv, 4002 Plovdiv, Bulgaria; 6 Department of Dental Prosthetics, Faculty of Dental Medicine, Iuliu Hatieganu University of Medicine and Pharmacy, 400083 Cluj-Napoca, Romania

**Keywords:** periodontitis, altered miRNA pattern, biological networks

## Abstract

In recent years, the role of microRNA (miRNA) in post-transcriptional gene regulation has advanced and supports strong evidence related to their important role in the regulation of a wide range of fundamental biological processes. Our study focuses on identifying specific alterations of miRNA patterns in periodontitis compared with healthy subjects. In the present study, we mapped the major miRNAs altered in patients with periodontitis (n = 3) compared with healthy subjects (n = 5), using microarray technology followed by a validation step by qRT-PCR and Ingenuity Pathways Analysis. Compared to healthy subjects, 159 differentially expressed miRNAs were identified among periodontitis patients, of which 89 were downregulated, and 70 were upregulated, considering a fold change of ±1.5 as the cut-off value and *p* ≤ 0.05. Key angiogenic miRNAs (miR-191-3p, miR-221-3p, miR-224-5p, miR-1228-3p) were further validated on a separate cohort of patients with periodontitis versus healthy controls by qRT-PCR, confirming the microarray data. Our findings indicate a periodontitis-specific miRNA expression pattern representing an essential issue for testing new potential diagnostic or prognostic biomarkers for periodontal disease. The identified miRNA profile in periodontal gingival tissue was linked to angiogenesis, with an important molecular mechanism that orchestrates cell fate.

## 1. Introduction

MicroRNAs (miRNAs) are evolutionarily conserved small non-coding RNA molecules that are 18–25 nucleotides long. They play a crucial role in normal cellular physiology, ensuring fine modulation of gene expression at a post-transcriptional level. Moreover, they coordinate tissue repair in adults [1,2,3].

In the first years after their discovery, the main focus of the miRNA was on their role in homeostasis; recent studies have observed that the abnormal expression of miRNAs leads to dysregulation of cellular responses involved in the adaptive immune reactions related to chronic inflammatory disease [4,5]. The fact that miRNAs are small molecules responsible for targeting multiple genes that belong to the same regulatory network makes them ideal tools for drug delivery and tissue regeneration [6].

miRNA function is essential for periodontal health, and immunity might affect periodontal homeostasis [4,7,8,9]. The process of gingival injury is followed by immediate tissue regeneration and repair [10]. At the cellular level, tissue regeneration and repair are triggered by cellular differentiation, dedifferentiation, transdifferentiation and reprogramming at the injured area. Moreover, injury-inducible coding genes and the signaling networks involved at the site of tissue interruption are controlled by miRNAs [2,4,9,11]. In addition, the significant mechanisms of angiogenesis, proliferation, migration, and morphogenesis of endothelial cells are controlled by specific miRNAs in an endothelial-specific manner. miRNAs known to regulate angiogenesis in vivo are called angiomiRs [7,11,12].

Lately, the expression of miRNAs in tissues affected by periodontitis has been explored [13]. Periodontitis and peri-implantitis are two diseases widely spread among the adult population, known to be triggered by a pathogenic bacterium that alters the host’s immune and inflammatory response [14]. From a clinical point of view, these alterations are translated into inflammatory lesions of the connective tissue around teeth or implants, with progressive bone loss and increased probing depths with an accelerating pattern [15]. The ongoing homeostasis between the bone resorption matrix and the new bone formation matrix is altered [16,17]. 

Differences in gene and miRNA expression in parodontids can affect specific cellular processes [18,19]. The literature reveals that the expression of specific miRNAs differs between the damaged periodontal tissue and the healthy periodontium. For example, miR-142-3p and miR-146a could be conclusive markers for disease activity [8]. Although the precise mechanism that leads to different changes in miRNA levels is not fully understood, studies have proven that the expression of certain miRNAs, such as miR-146a and miR-146b, was significantly higher. In contrast, the expression of miR-155 was significantly lower in inflamed tissues than in healthy tissues [9].

Periodontal disease is fast becoming the most frequent oral cavity disease, and implant therapy has already become a standard treatment for edentulous patients. To reduce the possible complications of implant therapy, such as peri-implantitis, our study aimed to identify the miRNAs as biomarkers in patients suffering from periodontitis, with subsequent consequences for the success of implant therapy and regeneration techniques.

This study aimed to identify the altered miRNA signature and its implication in regulating key molecular mechanisms related to periodontal disease and then to discuss key challenges in translating this knowledge to the clinic and to propose novel biomarkers for validation. Combining the understanding of miRNA biology with cutting-edge technologies for gene expression analysis, such as microarray and validation of the obtained data by qRT-PCR, would help establish new miRNAs with potential biomarker properties for parodontids disease. As criteria for qRT-PCR validation, miRNAs were selected from the top 25 upregulated and downregulated and, simultaneously, those involved in angiogenesis.

## 2. Materials and Methods

**Patients cohorts.** This study included 57 patients, 29 females (50.87%) and 28 males (49.13%). We had frozen gingival tissue for all the patients in the study, for microarray study are presented in Table 1 and for validation study in Table 2. The latest staging and classification in the field were used for the periodontitis diagnosis, according to the data published in 2018 [20]. The present study was approved by the institutional ethics committee of Iuliu Hatieganu University of Medicine and Pharmacy (UMPh), no. 81, from 11 March 2019.

**RNA extraction.** Fresh frozen tissue was used for RNA extraction using the classical phenol–chloroform method. Mainly, the tissue was homogenized in 800 µL TripleXtractor (Grisp, Portugal), and then, the sample was used for RNA extraction. First, the sample was treated with chloroform (160 µL), mixed well by vortex, incubated at room temperature (RT) for 5 min, and centrifuged for 20 min at 13,000 rpm and 4 °C. The transparent phase was transferred to a new 1.5 mL tube, and RNA was precipitated with 500 µL of isopropanol, mixed by tube inversion, and incubated for 15 min at RT, then centrifuged at 13,000 rpm and 4 °C for 15 min. The supernatant was removed, and the pellet was washed with 1 mL of 75% ethanol and centrifuged for 5 min at 10,000 rpm and 4 °C. After removal of the ethanol, the pellet was left to air-dry for 10–15 min and then dissolved in 25 µL nuclease-free water. The obtained RNA was quantified using NanoDrop (Thermo Fischer) spectrophotometer. 

**Gingival tissue miRNA microarray evaluation**. To evaluate the gingival miRNA pattern in both periodontitis and healthy tissues, 100 ng of total RNA from each sample was hybridized using a microRNA Spike-In kit and miRNA Complete Labeling and Hyb Kit (Agilent technologies). The microarray slides were hybridized for 20 h at a temperature of 55 °C, and washed and scanned using an Agilent Microarray Scanner. An additional purification step was performed using Micro Bio-Spin 6 (Biorad, Mississauga, ON, Canada) spin columns, followed by a desiccation step in a vacuum centrifuge and resuspension using 18 μL of RNase-free water.

**Microarray bioinformatics analysis**. Data analysis for each file was performed using the Agilent GeneSpring GX software. The obtained images were further processed using the Feature Extraction program from Agilent to convert them to tabular structures containing numeric values referring to the specific expression for each miRNA. After normalization, differential expression analysis was conducted using the “Filter on Volcano Plot” module, with moderated *t* test, a fold change cut-off of 1.5 and a *p* value < 0.05. The lists of differentially expressed miRNAs were exported from the software for subsequent analysis.

A series of comparisons were then performed using Venn diagrams to identify the miRNAs involved in specific signaling pathways such as angiogenesis and epithelial-to-mesenchymal transition. For this, we previously searched and downloaded from the NCBI (The National Center for Biotechnology Information) website the lists of genes involved in the pathways mentioned above by searching the Gene module for “Homo sapiens and angiogenesis” and “Homo sapiens and epithelial to mesenchymal transition”. 

**miRNA-mRNA network analysis**. The Ingenuity Pathway Analysis (IPA) software (Ingenuity Systems, Redwood City, CA, USA) analyzed miRNA upstream regulators, networks, and associated pathways. All altered miRNAs were integrated into networks and were algorithmically generated based on their connectivity and scores. The score is displayed as a numerical value considering the relevance of a particular network to the original list of transcripts.

**qRT-PCR validation of selected miRNA.** To evaluate the expression of miR-191-3p, miR-221-3p, miR-224-5p and miR-1228-3p, the obtained RNA was reverse transcribed using the TaqMan MicroRNA Transcription kit (Applied Biosystems) and TaqMan microRNA primer assay (ThermoFisher Scientific) for the selected miRNAs. RNU6 and RNU48 were used for data normalization. One µL of total RNA was mixed with 0.75 µL of 10X RT Buffer, 0.1 µL of RNase inhibitor, 0.075 µL dNTP, 0.1825 µL of each of the 20X miRNA RT primers, 4.52 µL of nuclease-free water, and 0.5 µL of MultiScribed RT enzyme. The mixture was incubated at 16 °C for 30 min, 42 °C for 30 min, 85 °C for 5 min and held at 4 °C. The obtained cDNA was diluted six times with nuclease-free water and then used in real-time PCR reaction. We made a mixture containing 5.03 µL of ready-to-use TaqMan Fast Advanced Master Mix (Applied Biosystems), 0.47 µL of TaqMan microRNA primer and 5.2 µL of cDNA for each of the miRNAs analyzed. Then, 5 µL of the ready mix was loaded into two individual wells of the PCR plate. The PCR program run on the Viia7 instrument was as follows: 1 cycle for 2 min at 50 °C, one cycle for 20 s at 95 °C and 40 cycles at 95 °C for 1 s and 60 °C for 20 s in FastMode. The obtained C_T_ values were analyzed using the ΔΔC_T_ method, and the obtained results were imported into GraphPad Prism software for graphical presentation. GraphPad Prism software performed the ROC (receiver operating characteristic curve) analysis. In contrast, multiROC curve analysis was performed using Combiroc web software (http://combiroc.eu/, accessed on 7 January 2023) to emphasize the diagnostic power [21] of miRNAs as biomarkers in parodontids.

## 3. Results

**Figure 1:**
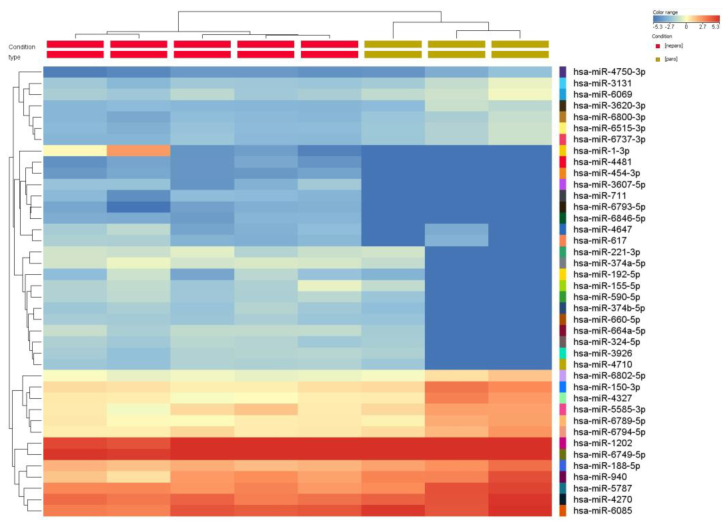
**Heatmap of the miRNA expression profile of periodontitis patients (n = 3) compared with healthy subjects (n = 5).** The extracted RNA from gingival tissue was fluorescently labeled, hybridized and analyzed by miRNA microarray. The obtained data were processed by Gene Spring software based on the hierarchical clustering of the analyzed samples. Red or green colors indicate differentially up- or downregulated miRNAs using FC ± 1.5 and *p* < 0.05 as cut-off values.

**Figure 2:**
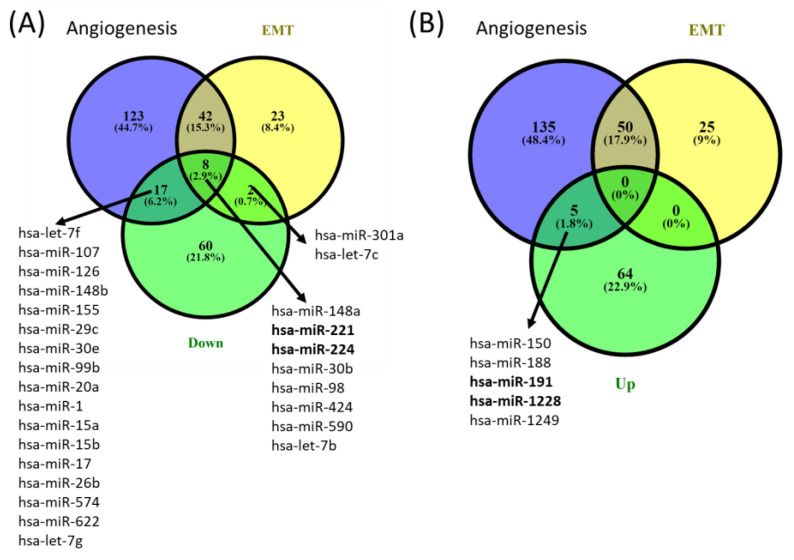
Venn diagrams of (**A**) downregulated and (**B**) upregulated angiogenesis and EMT overlapping miRNA signature from gingival samples with periodontal diseases compared to controls. The miRNA gene list was downloaded from the NCBI (The National Center for Biotechnology Information) website by searching the Gene module for “Homo sapiens and angiogenesis” and “Homo sapiens and epithelial to mesenchymal transition”.

**miRNA profiling in periodontitis and healthy gingival tissue.** miRNA microarray analysis using Agilent technology was used to uncover the miRNA expression profiles in samples from patients with periodontal disease (n = 3) and healthy controls (n = 5). The miRNAs with significant changes in expression level (fold change ±1.5, and *p* values < 0.05) are presented as a heatmap in Figure 1. Furthermore, 159 differentially expressed miRNAs were identified among patients with periodontitis compared with healthy subjects. Among them, 89 were downregulated, and 70 were upregulated (Table 3). By overlapping the altered miRNA signature with the miRNA related to angiogenesis and EMT (epithelial to mesenchymal transition) (downloaded from NCBI), we identified a panel of 8 miRNAs related to angiogenesis and EMT, 2 miRNAs associated with EMT, and 17 miRNAs related to angiogenesis from the downregulated miRNAs list in periodontitis. Five were related to angiogenesis from the overexpressed miRNA list (Figure 2).

**Network analysis by IPA**. IPA knowledge base was used to connect networks resulting in large merged networks. Predicted targets for the altered miRNAs list were mapped to the corresponding target in the Ingenuity knowledge base. The molecular and cellular functions identified based on altered miRNA signature are presented in Table 4.

**Figure 3:**
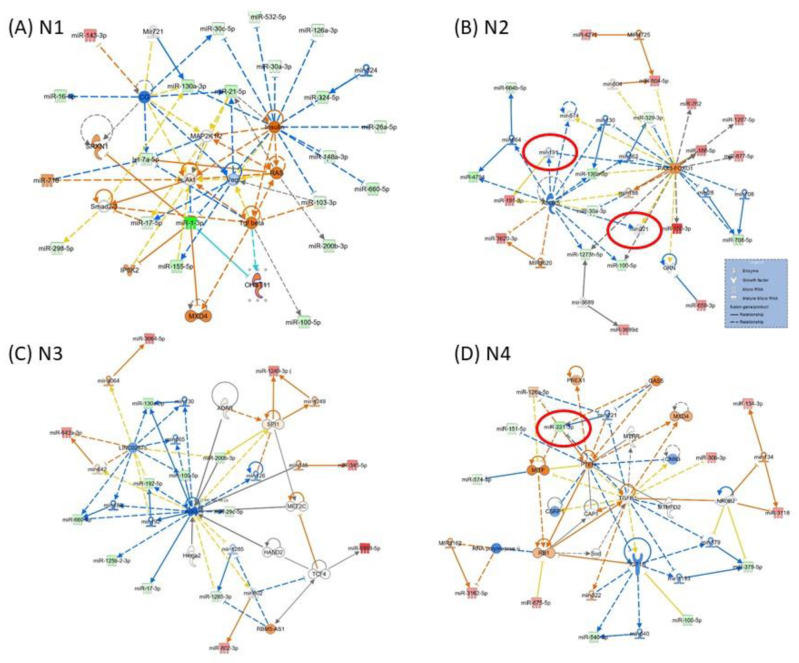
**Representative IPA network to predict the targets of altered miRNAs** (**A**) N1: Neurological Disease, Organismal Injury and Abnormalities, Psychological Disorders; (**B**) N2: Gene Expression, Organismal Injury and Abnormalities, Reproductive System Disease; (**C**) N3: Glomerular Injury, Inflammatory Disease, Inflammatory Response; (**D**) N4: Cellular Development, Cellular Movement, Protein Synthesis. The red color of molecules represents upregulated transcripts, whereas the green color represents downregulated transcripts in periodontal gingival tissue. The intensity of each color shows the strength of regulation. The solid arrows represent experimentally examined associations. A dashed line depicts the experimentally proven interactions. Solid feedback circular line with a hand is related to autoregulation.

Figure 3 presents the miRNA-mRNA network; in the case of network N1 (Figure 3A) being related to “Neurological Disease, Organismal Injury and Abnormalities, Psychological Disorders”, the core element is VEGF, interconnected with AKT, MAP2K1/2, SMAD2/3, TGFB and RAS. N2 (Figure 3B) is related to “Gene Expression, Organismal Injury and Abnormalities, Reproductive System Disease”, and the core elements of these networks are ALOX5 and PAX3-FOXO1. N3 (Figure 3C) is related to “Glomerular Injury, Inflammatory Disease, Inflammatory Response”, the core element of the network being AGO2. N4 is related to “Cellular Development, Cellular Movement, Protein Synthesis”, with the core genes represented by IGF1B, TGFB1 and PTEN; miR-221 downregulated in parodontids is directly interconnected with PTEN, GAS5 and MTIF. As modulated by miRNAs, these genes could be key signaling molecules in the network. The network function would change as a consequence, considering the high number of downregulated miRNAs interconnected with these genes.

**qRT-PCR validation of microarray data**. To validate the differentially expressed miRNAs from the microarray experiment, two downregulated and two overexpressed from the top 25 up/downregulated miRNAs were selected. The selection was based on their role in angiogenesis and the lack of data in the literature for their participation in periodontal disease. On the base of the above-applied, miR-191-3p, miR-221-3p, miR-224-5p and miR-1228-3p were chosen for validation by qRT-PCR, selected from the top 25 up-regulated and downregulated genes list and in the same time being related with the angiogenesis mechanism.

**Figure 4:**
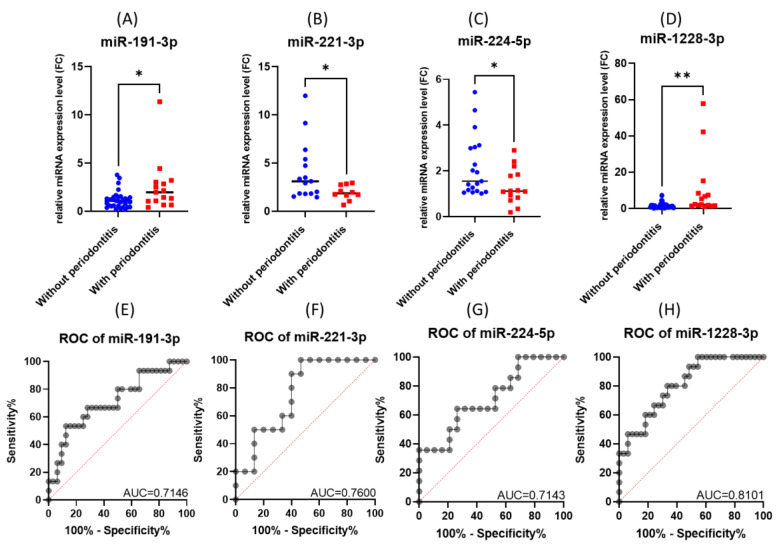
The expression level and ROC curve of tested miRNAs: (**A**) miR-191-3p, (**B**) miR-221-3p, (**C**) miR-224-5p, (**D**) miR-1228-3p. ROC curves for (**E**) miR-191-3p, (**F**) miR-221-3p, (**G**) miR-224-5p, (**H**) miR-1228-3p (* *p* < 0.05, ** *p* < 0.01).

The performed validation experiment confirmed the upregulation of miR-191-3p and miR-1228-3p and the downregulation of miR-221-3p and miR-224-5p in gingival periodontal tissue compared to normal gingiva tissue, a scenario observed in the microarray study (Figure 4A–D).

**Figure 5:**
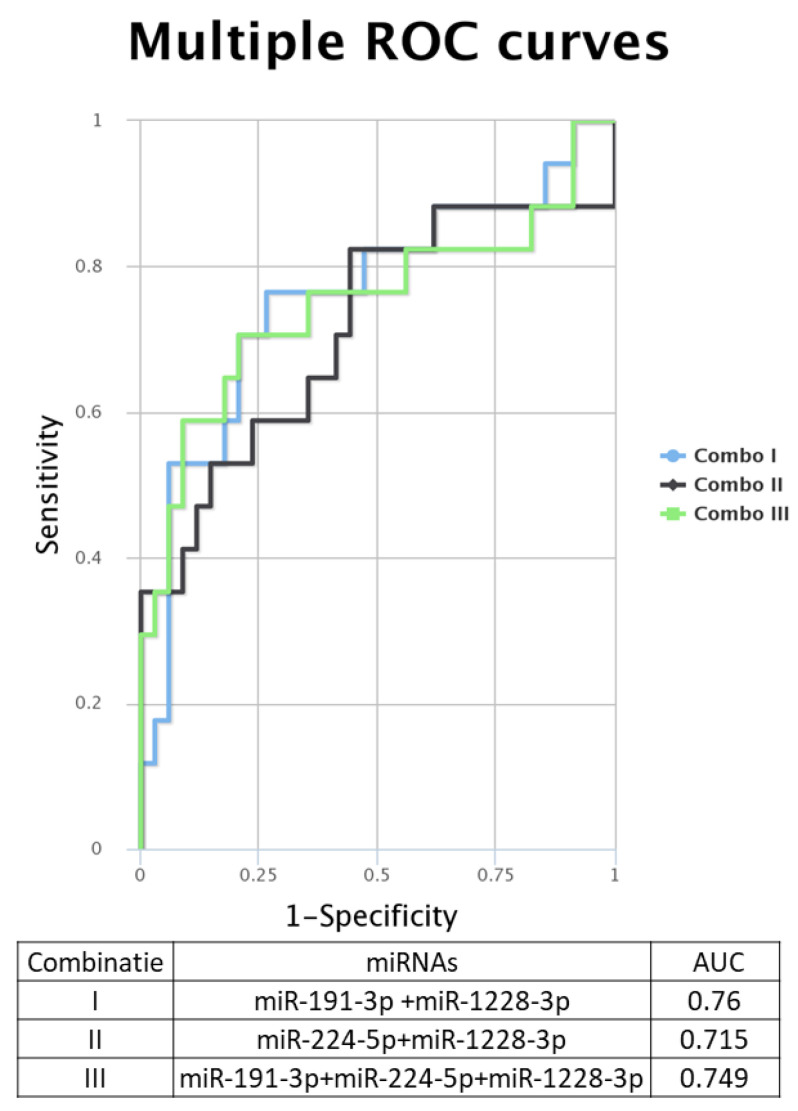
**Multiple ROC curves for the analyzed miRNA were generated using the online tool CombiROC**, for different combinations: Combo I: miR-191-3p + miR-1228-3p, Combo II: miR-224-5p + miR-1228-3p, Combo III: miR-191-3p + miR-224-3p + miR-1228.

When analyzing the ROC (receiver operating characteristic) curves for the selected miRNAs, we observed that all tested had an AUC (area under the curve) greater than 0.7 (Figure 4E–H). The highest AUC value (0.076) was observed for Combo I (miR-191-3p + miR-1228-3p) (Figure 5). Regarding the ROC curves for the combination of the tested biomarkers, we obtained only ROC curves for three of the four biomarkers tested, which passed the quality control criteria for specificity and selectivity of the CombiROC software.

Table 5 presents the statistical values using Pearson correlation between the expressions of the tested targets. The values in red represent statistically significant correlations. A direct statistically significant correlation was observed between miR-221-3p and miR-224-5p and between miR-224-5p and miR-1228-3p.

**Figure 6:**
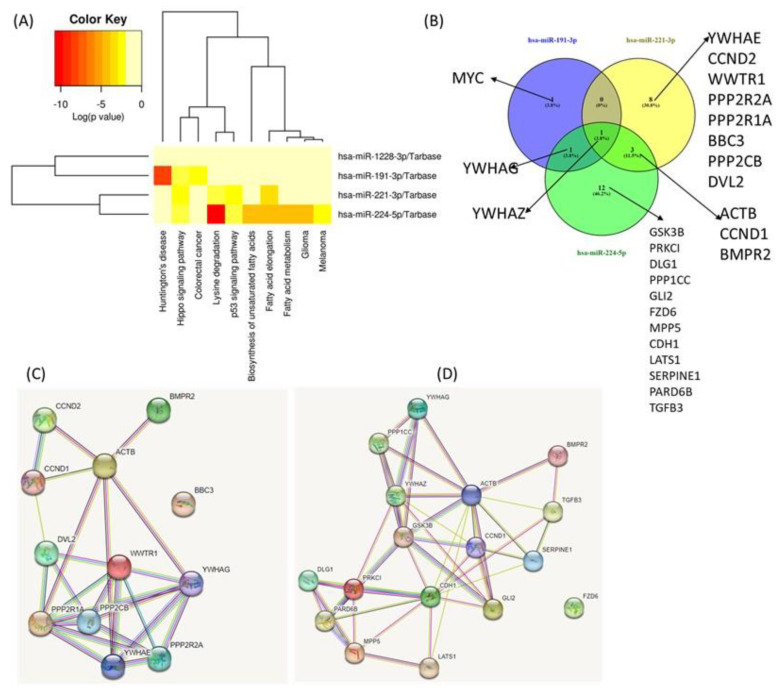
**Functional Analysis of miRNAs Using DIANA-miRPath v3.0 interface**. (**A**) Heatmap created directly from the DIANA-miRPath v3.0 interface. The heatmap depicts the level of enrichment in GO categories of miR-191-3p, miR-221-3p, miR-224-5p and miR-1228-3p. (**B**) Venn Diagram of main miRNA targets related to hippo signaling. (**C**) protein network generated based on the main targets connected to miR-221-3p. (**D**) Protein network generated based on the main targets related to miR-224-5p.

**Complex biological processes modulated by miR-191-3p, miR-221-3p, miR-224-5p and miR-1228-3p and their target genes**. The main physical processes and target genes for miR-191-3p, miR-221-3p, miR-224-5p and miR-1228-3p were identified using the DIANA-miRPath v3.0 interface. A heatmap created directly from the DIANA-miRPath v3.0 interface reveals the main biological pathways that the selected miRNAs target (Figure 6A). Thus, we identified several overlapping target genes related to hippo signaling between the selected miRNAs, as follows: for miR-191-3p, three target genes; for miR-221-3p, twelve target genes; and for miR-224-5p, sixteen target genes were observed. Only miR-1228-3 had no target gene overlapping with the others. These data could emphasize YWHAZ as a common target for the first three transcripts (Figure 6B). Given the intricacy of the regulation of processes touched by these transcripts, predicting the dynamics of gene expression in parodontids is challenging. Additional functional studies will be required to evaluate the relative contribution of individual players.

## 4. Discussion

In the present study, we conducted a miRNA microarray analysis of gingival tissue for patients with periodontitis versus healthy tissue and further validated it in independent validation sets. We identified a panel of potential novel biomarker candidates with application in periodontal disease [8,13], with implications in dental implantation for these patients. The periodontal region is a highly dynamic microenvironment that undergoes continuous remodeling due to frequent tissue fitting mechanical stress and inflammatory conditions.

miRNAs are considered promising candidates based on their better features, including high abundance, stability, ease of sampling, and importance as global cellular regulators. The evaluation of the expression differences in miRNA and miRNA-mRNA interactions is only available in a few studies on periodontal disease. They can target several genes and influence multiple regulatory networks [22], also sustained by IPA analysis. Understanding the expression variation may help us deduce the occurrence and development of this complex disease (Figure 3).

Previously, an IPA analysis revealed that the altered miRNA pattern in parodontids in the Japanese population is associated with inflammatory disease, organismal injury, abnormalities, urological disease, and cancer [23], a fact confirmed by the present data.

The literature presents VEGF as a potential molecular target in periodontitis [24]. This is the core element of N1 (Figure 3A), directly interconnected by miR-21 and miR-1. MiR-21 is upregulated in patients with periodontitis and in mice induced with a periodontitis field [14,17], confirmed by the present microarray data. The literature data present that the FOXO1 signaling axis can regulate periodontal bacteria–epithelial interactions, immune-inflammatory response, bone remodeling, and wound healing [25]. In N2 from Figure 3B, it emphasizes the interconnection of the altered miRNAs with PAX3-FOXO1. Several therapeutic approaches targeting PAX3-FOXO1 were developed [26], the axis that should be further considered and exploited for periodontal disease.

miRNAs act as post-transcriptional gene suppressors through their association with argonaute 2 (AGO2), a vital member of the RNA induced silencing complex (RISC) [27]. Several studies have demonstrated miRNA interaction with AGO2 [27]. AGO2 is crucial for the biogenesis of miRNAs and functions of multiple mechanisms, including angiogenesis [28,29]. AGO2 is considered a marker of differentiating periodontitis. AGO2 is the core of N3, interconnected with several downregulated miRNAs in parodontids.

Our study reveals that periodontal diseases are more complex than previously assumed, emphasizing the inhibition of an important number of angiogenic-related miRNAs, as shown in Figure 2. We identified five angiogenesis-related miRNAs; in the top 25 upregulated, there were only four (miR-150, miR-188, miR-191 and miR-1228). Among these transcripts, there are limited data related to the implication in periodontitis; one study presented miR-150, miR-223 and miR-200b as overexpressed and miR-379, miR-199a-5p and miR-214 as underexpressed in inflamed gingival tissues in a Japanese population [23]. miR-188-3p was demonstrated to suppress human periodontal ligament stem cell osteogenesis through upregulating LEP [30]. No other information is related to the implication of miR-191 and miR-1228 in parodontids. Only 3 of the top 25 downregulated miRNAs (miR-221, miR-224 and miR-540) were related to angiogenesis and EMT, with none of these transcripts known to be related to periodontitis.

Our findings clearly emphasized the clinical utility of miR-191-3p, miR-221-3p, miR-224-5p and miR-1228-3p when analyzed individually or when considered as the signature in their ability to efficiently distinguish periodontitis patients from controls. Moreover, as we analyzed samples from periodontitis regions, we could speculate that miR-191-3p, miR-221-3p, miR-224-5p and miR-1228-3p expression is disease-specific. These findings suggest that periodontitis-specific miR-191-3p, miR-221-3p, miR-224-5p and miR-1228-3p modulation is a new molecular tool for disease diagnosis, which can be validated through further studies on different and larger patient populations.

One of the miRNAs identified in our study, miR-221-3p, appeared to be induced in mechanical force-induced osteoblastic/cementoblasts differentiation of human periodontal ligament cells, thus providing a direct link of the above-mentioned process with the development of periodontitis [31]. In addition, Qiao et al. reported that miR-224-5p is highly expressed in dental periodontal ligament cells compared to dental pulp stem cells. Further functional studies performed on miR-221-3p-depleted dental pulp stem cells showed impaired cell viability followed by apoptosis through Rac family small GTPase 1 (Rac1) direct targeting [32].

miR-1228 was previously described as an actor of osteoblastic cell differentiation, targeting BMP-2K (bone morphogenetic protein–2 induced kinase) by inhibiting protein translation [33].

Aravindraja et al. proved that differentially expressed miRNAs that target different pathways in periodontal disease are connected to bacterial invasion and host response. They mention that miR-191 is involved in ischemic stroke [34], which leads to the idea that since periodontal disease is proven to be related to heart disease [35], miR-191 should also be investigated more closely in the oral field, as we stated above.

Hippo signaling is an important pathway involved that affects mineralized tissue homeostasis and remodeling [36,37]. Our study emphasizes the diverse target genes related to Hippo signaling to the validated miRNAs. This should be further investigated for deciphering complex regulatory pathways related to miR-191-3p, miR-221-3p and miR-224-5p. miR-191-3p is not presented in the literature as related to parodontids disease. miR-221-3p modulates apoptosis in periodontal ligament cells [38]. miR-221-3p and miR-222-3p inhibited osteogenic differentiation of BMSCs via the IGF-1/ERK pathway [38]. Downregulation of miR-224-5p may promote dental pulp stem cell proliferation and migration [39].

As future perspectives, an additional investigation should be considered to better understand the altered mechanisms, which will lead to better therapeutic strategies for parodontids, particularly when considering the limitation of our study related to the reduced number of cases used for the microarray and for the validation cohort by qRT-PCR.

Our research is focused on miRNA analysis as a potential biomarker for periodontal disease, with subsequent benefits in clinical protocols, and it involves highly trained specialists and financial resources. We consider it useful to identify patients at risk by using chairside point-of-care diagnostic technologies (PoCT) such as the aMMP-8 (active matrix metalloproteinase-8) oral fluid test as a first step in selecting more proper patients who require deeper and more complex investigations before implant placement [40]. Future research is required to validate the mechanism of these transcripts to potentially benefit from the angiogenic-related miRNAs’ roles in implant regeneration therapy.

## 5. Conclusions

Our findings indicate an altered pattern of miRNA in gingival tissue, which should be considered an essential issue in generating new prognostic or diagnostic biomarkers for periodontal diseases, such as miR-191-3p, miR-221-3p, miR-224-5p and miR-1228-3p.

Understanding the functional roles of miRNAs in the pathogenesis of periodontitis is very important due to their strong potential as therapeutic targets in alveolar bone regeneration. miRNAs display a specific periodontal gingival tissue, indicating that alterations in angiogenesis and critical cellular signaling networks have important implications in periodontal homeostasis and disease. Therefore, it is crucial to elucidate further how these altered transcripts might play a role in periodontal homeostasis and illness, considering the complex molecular mechanisms regulated by the altered transcripts.

## Figures and Tables

**Table 1:** Patient selection for the microarray analysis.

Patient NO.	Tissue Sample Gingival Tissue (GT)	Age (Years)	Gender (Female/Male)	Control Group	Periodontitis
12.	GT 16	44	Female	+	
13.	GT 17	64	Female		+
16.	GT 22	25	Male	+	
22.	GT 30	35	Male		+
34.	GT 44	33	Female	+	
35.	GT 45	59	Male	+	
38.	GT 48	31	Female	+	
46.	GT 58	69	Male		+

**Table 2:** Patient data validated through qRT-PCR.

Patient NO.	Tissue Sample Gingival Tissue (GT)	Age (Years)	Gender (Male/Female)	Periodontitis
1.	GT 1	48	Female	
2.	GT 2	35	Male	
3.	GT 4	25	Male	
4.	GT 5	34	Female	
5.	GT 6	48	Female	+
6.	GT 8	36	Male	
7.	GT 9	64	Male	+
8.	GT 10	37	Female	
9.	GT 11	35	Female	
10.	GT 12	41	Male	
11.	GT 14	38	Male	+
14.	GT 18	50	Male	+
15.	GT 21	34	Female	
17.	GT 25	38	Male	
18.	GT 26	39	Male	
19.	GT 27	56	Male	
20.	GT 28	32	Male	
21.	GT 29	30	Female	
23.	GT 31	74	Female	+
24.	GT 34	47	Female	+
25.	GT 32	31	Female	
26.	GT 33	49	Female	+
27.	GT 35	43	Female	
28.	GT 37	58	Male	+
29.	GT 38	51	Female	
30.	GT 40	67	Male	+
31.	GT 41	66	Female	+
32.	GT 42	64	Male	+
33.	GT 43	45	Female	
36.	GT 46	67	Female	
37.	GT 47	38	Female	
39.	GT 49	51	Female	
40.	GT 50	44	Female	+
41.	GT 51/GT 52	31	Male	+
42.	GT 53	60	Female	
43.	GT 54/GT 55	31	Male	+
44.	GT 56	34	Male	+
45.	GT 57	53	Male	
47.	GT 59	40	Male	
48.	GT 60/GT 61	40	Male	+
49.	GT 62	32	Male	
50.	GT 63	51	Female	
51.	GT 64	60	Female	
52.	GT 65	44	Male	
53.	GT 66	54	Female	
54.	GT 67	44	Male	
55.	GT 68/GT 69	45	Female	+
56.	GT 70	48	Female	
57.	GT 71/GT 72	38	Female	

**Table 3:** miRNA expression profiles in periodontal disease gingival tissue (n = 3) and healthy controls (n = 5), considering fold change (FC) ± 1.5 and *p* < 0.05 as cut-off values.

miRNA Name	FC	*p* Value
hsa-miR-1-3p	−34.12857	0.037032157
hsa-miR-3607-5p	−18.97933	4.09 × 10^−4^
hsa-miR-374a-5p	−16.82007	0.022104776
hsa-miR-711	−15.7409	3.89 × 10^−4^
hsa-miR-6846-5p	−14.31281	1.37 × 10^−4^
hsa-miR-664a-5p	−12.49932	0.019430375
hsa-miR-221-3p	−12.4242	0.04215776
hsa-miR-590-5p	−10.74705	0.020470627
hsa-miR-6793-5p	−10.37311	0.001994425
hsa-miR-155-5p	−10.25699	0.048597593
hsa-miR-454-3p	−9.837945	2.28 × 10^−4^
hsa-miR-4481	−9.251524	3.43 × 10^−4^
hsa-miR-4647	−9.232709	0.026655879
hsa-miR-324-5p	−9.203884	0.034039732
hsa-miR-192-5p	−9.176847	0.02487085
hsa-miR-374b-5p	−9.13536	0.0221324
hsa-miR-4710	−8.932179	0.030074801
hsa-miR-617	−8.825279	0.0285748
hsa-miR-3926	−8.810145	0.041111533
hsa-miR-660-5p	−8.539844	0.027984386
hsa-miR-224-5p	−8.425938	0.04408213
hsa-miR-6747-5p	−8.419437	0.04841601
hsa-miR-379-5p	−8.364333	6.48 × 10^−4^
hsa-miR-99b-5p	−8.296491	0.041821264
hsa-miR-28-5p	−8.215269	0.04408498
hsa-miR-424-5p	−8.137206	0.03456241
hsa-miR-429	−8.068692	0.049520656
hsa-miR-130b-3p	−7.767458	0.040380087
hsa-miR-98-5p	−7.765576	0.01406511
hsa-miR-4538	−7.741504	0.027376672
hsa-miR-5684	−7.628777	0.006664117
hsa-miR-365a-5p	−6.989398	0.008685829
hsa-miR-1273h-5p	−6.657006	0.04518406
hsa-miR-6801-5p	−6.651721	0.014889749
hsa-miR-6890-5p	−6.53072	0.03981066
hsa-miR-766-3p	−6.495997	0.044748943
hsa-miR-6777-5p	−6.451282	0.027593505
hsa-miR-6776-5p	−6.408142	0.032330293
hsa-miR-4419b	−6.247995	0.029865561
hsa-miR-7854-3p	−6.231512	0.03727845
hsa-miR-3147	−6.202302	0.021174427
hsa-miR-4749-5p	−5.920589	0.04640766
hsa-miR-6511a-5p	−5.758417	0.0434213
hsa-miR-622	−5.720877	0.013109049
hsa-miR-7515	−5.700151	0.044666458
hsa-miR-1471	−5.678476	0.049022842
hsa-miR-301a-3p	−5.667045	0.02680192
hsa-let-7e-5p	−5.577964	0.01425882
hsa-miR-6870-5p	−5.446335	0.026244618
hsa-miR-662	−5.396921	0.03185127
hsa-miR-6796-5p	−5.303267	0.035478767
hsa-miR-4436b-5p	−5.275263	0.0323301
hsa-miR-5190	−5.241223	0.041012246
hsa-miR-148b-3p	−5.222345	0.023750385
hsa-miR-4728-5p	−5.157973	0.039344892
hsa-miR-550a-3-5p	−5.155397	0.015180632
hsa-miR-17-3p	−5.122168	0.048030578
hsa-miR-532-5p	−5.074659	0.030368464
hsa-miR-1285-3p	−4.999519	0.02915273
hsa-miR-4449	−4.938702	0.04064014
hsa-miR-30e-3p	−4.749875	0.045240145
hsa-let-7d-5p	−4.729961	0.011936195
hsa-let-7f-5p	−4.628874	0.009347751
hsa-miR-2467-3p	−4.511706	0.0329003
hsa-miR-224-3p	−4.249713	0.040188752
hsa-miR-29c-5p	−3.785163	0.03951475
hsa-let-7a-5p	−3.774148	0.010737276
hsa-miR-4462	−3.672874	0.030366512
hsa-miR-6792-5p	−3.645321	0.043304242
hsa-let-7g-5p	−3.414934	0.013226924
hsa-miR-362-3p	−3.318821	0.040582217
hsa-let-7i-5p	−3.067374	0.016266054
hsa-miR-17-5p	−2.884122	0.044331945
hsa-miR-20a-5p	−2.791418	0.032910347
hsa-miR-15b-5p	−2.682962	0.048703965
hsa-miR-26b-5p	−2.676988	0.006512697
hsa-miR-16-5p	−2.572164	0.025805915
hsa-miR-664b-5p	−2.524908	0.008731748
hsa-miR-151a-5p	−2.424799	0.026036028
hsa-miR-15a-5p	−2.366632	0.01141522
hsa-miR-126-3p	−2.254291	0.041778404
hsa-miR-103a-3p	−2.175419	0.03020916
hsa-miR-107	−2.169281	0.021729073
hsa-miR-148a-3p	−2.104684	0.009180019
hsa-miR-574-5p	−2.095274	0.01953186
hsa-let-7c-5p	−2.087441	0.022676412
hsa-miR-30b-5p	−2.00868	0.04326747
hsa-let-7b-5p	−1.921514	0.03869098
hsa-miR-1973	−1.534013	0.021931823
hsa-miR-4327	3.245765	0.028274259
hsa-miR-150-3p	3.242801	0.02167679
hsa-miR-940	3.036692	0.036002662
hsa-miR-1202	2.606347	0.010853041
hsa-miR-6749-5p	2.534547	0.014533655
hsa-miR-5787	2.378687	0.045871302
hsa-miR-5585-3p	2.257536	0.04775345
hsa-miR-6789-5p	2.214927	0.045032978
hsa-miR-6085	2.210305	0.03939112
hsa-miR-6794-5p	2.13643	0.025145033
hsa-miR-188-5p	2.086498	0.014580581
hsa-miR-6069	2.078372	0.014913113
hsa-miR-4750-3p	2.074188	0.045291472
hsa-miR-3131	2.07223	0.03601266
hsa-miR-6800-3p	2.061477	0.010229712
hsa-miR-6737-3p	2.039422	0.010190696
hsa-miR-3620-3p	2.023533	0.021724554
hsa-miR-4270	2.020551	0.021035919
hsa-miR-6802-5p	2.017505	0.02563477
hsa-miR-6515-3p	2.009278	0.011002767
hsa-miR-1238-3p	2.000145	0.009585262
hsa-miR-6508-5p	1.999633	0.04351108
hsa-miR-191-3p	1.992056	0.033258095
hsa-miR-1228-3p	1.987597	0.014103335
hsa-miR-4687-3p	1.982589	0.030141398
hsa-miR-1225-5p	1.978492	0.025724296
hsa-miR-1249-3p	1.977446	0.01535282
hsa-miR-6165	1.923403	0.016845224
hsa-miR-762	1.914292	0.014265048
hsa-miR-6088	1.911795	0.009287248
hsa-miR-4787-3p	1.907168	0.016863897
hsa-miR-8069	1.906678	0.023480447
hsa-miR-6819-3p	1.895276	0.019375492
hsa-miR-601	1.883773	0.008287855
hsa-miR-642a-3p	1.873261	0.016594449
hsa-miR-6724-5p	1.868632	0.033642147
hsa-miR-4763-3p	1.860797	0.046400476
hsa-miR-4472	1.860704	0.031152643
hsa-miR-4725-5p	1.852989	0.038346846
hsa-miR-659-3p	1.814817	0.011700504
hsa-miR-3656	1.782478	0.029946806
hsa-miR-3960	1.779619	0.011423088
hsa-miR-3937	1.766218	0.02473999
hsa-miR-3194-5p	1.757172	0.01325334
hsa-miR-6786-5p	1.756058	0.019267108
hsa-miR-4741	1.748406	0.001054266
hsa-miR-4433a-5p	1.740275	0.028125772
hsa-miR-5739	1.700487	0.04004633
hsa-miR-6891-5p	1.683645	0.007658964
hsa-miR-6851-5p	1.67671	0.022706749
hsa-miR-642b-3p	1.676616	0.047492933
hsa-miR-4745-5p	1.675799	0.035927374
hsa-miR-1207-5p	1.67196	0.020776762
hsa-miR-3162-5p	1.661026	0.0104382
hsa-miR-877-5p	1.654778	0.020883325
hsa-miR-134-5p	1.6407	0.017721886
hsa-miR-4721	1.612612	0.004277528
hsa-miR-4466	1.604304	0.045366645
hsa-miR-1268a	1.594011	0.024818288
hsa-miR-4740-5p	1.593275	0.018724632
hsa-miR-4271	1.580986	0.018550621
hsa-miR-6728-5p	1.574383	0.01933102
hsa-miR-6893-5p	1.565816	0.031139249
hsa-miR-6813-3p	1.55972	0.013829393
hsa-miR-6800-5p	1.543613	0.0461837
hsa-miR-30d-5p	1.542779	0.035506163
hsa-miR-4322	1.537713	0.04705109
hsa-miR-4313	1.536664	0.023304904
hsa-miR-937-5p	1.525383	0.041646298
hsa-miR-8089	1.512869	0.026609493

**Table 4:** Top molecular and cellular functions identified based on altered miRNA signature.

Biological Process	*p* Value Range	Number of Molecules
Cellular Movement	4.58 × 10^−2^–5.66 × 10^−7^	21
Cellular Development	4.79 × 10^−2^–1.01 × 10^−6^	29
Cellular Growth and Proliferation	4.79 × 10^−2^–1.01 × 10^−6^	30
Cell Cycle	4.58 × 10^−2^–2.96 × 10^−5^	9
Cell Death and Survival	4.86 × 10^−2^–1.11 × 10^−4^	15

**Table 5:** Correlation using Pearson Correlation between the expressions of the tested targets.

*p* Value	miR-191-3p	miR-221-3p	miR-224-5p	miR-1228-3p
miR-191-3p		0.69	0.70	0.39
miR-221-3p	0.69		0.04	0.94
miR-224-5p	0.70	0.04		0.02
miR-1228-3p	0.39	0.94	0.02	

## Data Availability

Data are available on request.
